# Abstracts of the 2022 Canadian Association of Medical Oncologists Annual Scientific Meeting †

**DOI:** 10.3390/curroncol29060317

**Published:** 2022-05-31

**Authors:** Doreen Ezeife, Olexiy Aseyev, Sharlene Gill, Stephen Welch, Bruce Colwell, Desiree Hao

**Affiliations:** Canadian Association of Medical Oncologists, Ottawa, ON K1E 3R9, Canada; doreen.ezeife@albertahealthservices.ca (D.E.); aseyevo@tbh.net (O.A.); sgill@bccancer.bc.ca (S.G.); stephen.welch@lhsc.on.ca (S.W.); bruce.colwell@nshealth.ca (B.C.)

**Keywords:** medical oncology, cancer, research

## Abstract

On behalf of the Canadian Association of Medical Oncologists, we are pleased to present the abstracts of the 2022 Annual Meeting. The CAMO Virtual Annual Scientific Meeting (ASM) took place on 28 April 2022. Twenty-five (25) abstracts were selected for presentation as oral presentations and poster presentations. Awards for the top three (3) abstracts were presented during the ASM. All of them are marked as “Award Recipient”. We congratulate all the presenters on their research work and contribution.

**Award Recipient**

## 1. Oral Presentation

**Characterization of Immune-Related Adverse Events and Their Impact on Outcomes in Metastatic Melanoma (MM) Treated with Anti-PD-1 Alone or in Combination with Anti-CTLA-4 Immunotherapy**
Alexander Watson ^1^, Siddhartha Goutam ^2^, Igor Stukalin ^3^, Benjamin W Ewanchuk ^4^, Michael Sander ^4^, Daniel E Meyers ^3^, Aliyah Pabani ^1^, Winson Cheung ^1^, Daniel Heng ^1^, Tina Cheng ^1^, Jose G Monzon ^1^, Vishal Navani ^1^
^1^ Tom Baker Cancer Centre, Department of Oncology, University of Calgary, Calgary, AB, Canada
^2^ Faculty of Medicine and Dentistry, University of Alberta, Edmonton, AB, Canada
^3^ Department of Medicine, University of Calgary, Calgary, AB, Canada
^4^ Cumming School of Medicine, University of Calgary, Calgary, AB, Canada
* Correspondence: alexander.watson@albertahealthservices.ca

*Objective*

Immune-related adverse events (irAEs) due to immune checkpoint blockade (ICB) have been shown to correlate positively with survival outcomes in multiple solid tumors. For MM, data have been more conflicting, and combination ICB therapy with ipilimumab/nivolumab, classically associated with increased irAE toxicities, is supported by limited real-world evidence. We sought to characterize irAE and resulting outcomes using the Alberta Immunotherapy Database (AID).

*Methods*

The AID database is a multi-center, province-wide, retrospective dataset that includes consecutive patients treated with ICB. We included adult patients with MM from August 2013 to May 2020. Baseline characteristics between patients that developed significant irAEs (requiring systemic steroids and/or treatment delay) and those that did not were compared using the chi-squared test. To reduce immortal time bias, a 12-week landmark was applied for overall survival (OS), compared via log-rank test. Cox regression analyses were performed to determine the association of variables with OS.

*Results*

Of 492 MM patients, 364 received single-agent ICB (pembrolizumab or nivolumab), while 124 received combination ICB. A total of 87 patients (70.2%) on combination ICB experienced irAEs, and 35 (28.2%) required hospitalization, compared to 30.2% and 8.7%, respectively, for single ICB. IrAEs were more common in patients <50 yo (*p* = 0.02), with ECOG 0 (*p* < 0.001), while tumor characteristics were not associated. Patients with irAEs had longer median OS (56.31 vs. 18.45 mo, *p* < 0.0001), which was maintained in patients treated with combination ICB (median OS 56.30 vs. 19 mo, *p* < 0.0001). Type of irAE did not impact OS. Favourable association of irAE development with OS was confirmed in multivariable Cox analysis (HR 0.376, *p* < 0.001).

*Conclusions*

In the largest cohort of combination-ICB-treated patients described in a real-world setting to date, irAEs remain associated with survival outcomes, despite increased hospitalization. The building literature around this topic will help clinicians to advise and manage patients who experience irAEs.

**Award Recipient**

## 2. Oral Presentation

**Treatment of Relapsed Small-Cell Lung Cancer in the Real World: A Comparison of Platinum–Etoposide Rechallenge and Cyclophosphamide-Doxorubicin-Vincristine (CAV) Chemotherapy**
Sara Moore ^1^, Deepro Chowdhury ^1^, Mathieu Mckinnon ^2^, Paul Wheatley-Price ^1^
^1^ The Ottawa Hospital Research Institute, Ottawa, ON, Canada
^2^ University of Ottawa, Ottawa, ON, Canada
* Correspondence: smoore@toh.ca

*Objective*

Explore the use of second-line therapy for small-cell lung cancer (SCLC) in the real world, with a focus on comparing platinum–etoposide (PE) rechallenge to cyclophosphamide-doxorubicin-vincristine (CAV) chemotherapy.

*Methods*

We reviewed all patients diagnosed with SCLC at the Ottawa Hospital from January 2014 to December 2018 and identified those treated with second-line systemic therapy. Baseline demographics, diagnostic, and treatment information were collected. The primary outcome was overall survival after platinum rechallenge compared to CAV.

*Results*

During the study period, 539 patients were diagnosed with SCLC, 446 (83%) received first-line systemic therapy, and 106/446 (24%) went on to receive second-line systemic therapy. Baseline characteristics at initial diagnosis of this second-line cohort: median age 64.5, 60 (57%) female, 105 (99%) former/current smoking history, 90 (85%) extensive stage. First-line treatment was almost exclusively platinum-based (*n* = 105, 99%). At the time of second-line therapy: 73 (69%) had ECOG performance status (PS) 0–1, and 76 (72%) had chemosensitive disease with a treatment-free interval (TFI) > 90 days. Second-line regimens included platinum–etoposide (*n* = 48, 45%), CAV (*n* = 43, 41%), platinum–irinotecan (*n* = 4, 4%), docetaxel (*n* = 4, 4%), and other (*n* = 7, 7%). Median overall survival from the start of second-line chemotherapy was 6.2 months (m).

Comparing patients who received PE rechallenge versus CAV, there were no significant differences in baseline characteristics including age, sex, smoking history, stage at diagnosis, and ECOG PS. Patients receiving CAV were more likely to have a TFI < 180 days (81% vs. 52%, *p* = 0.004). PE chemotherapy led to higher physician-assessed clinical benefit (87% vs. 40%, *p* < 0.001) and overall survival (median OS 9.0 m versus 3.8 m) compared to those receiving CAV. In a sensitivity analysis, the survival difference persisted in patients with TFI < 180 days.

*Conclusions*

Patients who receive second-line systemic therapy for relapsed SCLC represent a small subset of the overall SCLC patient population. In this real-world study, platinum–etoposide rechallenge appeared to offer benefits over CAV, though there may be unrecognized confounding factors.

## 3. Oral Presentation

**The Impact of Virtual Oncology Care on Chemotherapy Continuity and Clinical Outcomes in Patients Treated for Colorectal Cancer during the COVID-19 Pandemic**
William J Phillips ^1^, Macyn Leung ^2^, Kednapa Thavorn ^2,3^, Timothy Asmis ^2,4^
^1^ Faculty of Medicine, University of Ottawa, Ottawa, ON, Canada
^2^ The Ottawa Hospital Research Institute, University of Ottawa, Ottawa, ON, Canada
^3^ University of Ottawa School of Epidemiology and Public Health, Ottawa, ON, Canada
^4^ The Ottawa Hospital Regional Cancer Centre, Ottawa, ON, Canada
* Correspondence: wiphillips@toh.ca

*Objective*

The coronavirus disease 2019 (COVID-19) resulted in an abrupt transition to virtual oncology care worldwide. This study’s objective was to evaluate the quality of systemic therapy in patients receiving chemotherapy for colorectal cancer before and during the COVID-19 pandemic.

*Methods*

This observational study evaluated patients treated with intravenous chemotherapy for colorectal cancer consecutively at the Ottawa Hospital Cancer Centre from June 2019 to September 2021. Non-metastatic rectal cancers were excluded. Patients were stratified by whether they started chemotherapy pre-pandemic (June 2019–January 2020) versus intra-pandemic (February 2020–September 2021). Baseline characteristics and treatment data were collected from electronic medical records. Outcomes of interest included chemotherapy delays, dose reductions, emergency department (ED) visits and hospitalizations. We used generalized linear and binary logistic regression modelling to compare outcomes between pre- and intra-pandemic periods.

*Results*

There were 220 patients included in this study, with 108 (49%) diagnosed with metastatic disease. In total, there were 66 (30%) patients treated in the pre-pandemic and 154 (70%) in the intra-pandemic period. Virtual consultations and follow-up visits increased during the pandemic from 1.5% to 43.5% (*p* < 0.001) and 37% to 84% (*p* < 0.001), respectively.

There was no difference in the incidence of treatment delays (OR = 1.01, *p* = 0.78), dose reductions (OR = 0.99, *p* = 0.69), ED visits (OR = 1.23, *p* = 0.37), hospitalizations (OR = 0.73, *p* = 0.43) or total length of treatment breaks (OR = 0.85, *p* = 0.17) between the pre- and intra-pandemic periods by multivariable analysis. Subgroup analysis showed no difference in outcomes independent of the presence of metastases.

*Discussion*

This study demonstrates no significant difference in chemotherapy dose intensity, continuity or clinical outcomes in patients treated for colorectal cancer during the COVID-19 pandemic. These findings serve as an important quality care indicator and suggest virtual oncology is safe in high-risk colorectal cancer patients on active, systemic therapy. Future work dedicated to other tumor sites would allow for broader application of these findings.

## 4. Oral Presentation

**Updated Data on the Role of Local Ablative Therapy for Oligoprogressive Disease in Oncogene-Addicted Non-Small-Cell Lung Cancer**
David Tsui ^1^, Douglas E Holt ^2^, Tejas Patil ^1^, D. Ross Camidge ^1^, Robert C Doebele ^1^, Paul A Bunn ^1^, Brian D Kavanagh ^2^, Jose Pacheco ^1^, William T Purcell ^1^, Erin Schenk ^1^, Chad R Rusthoven ^2^
^1^ Division of Medical Oncology, Department of Medicine, University of Colorado Cancer Center, Denver, CO, USA
^2^ Department of Radiation Oncology, University of Colorado Anschutz Medical Campus, Denver, CO, USA
* Correspondence: chuncheo@ualberta.ca

*Background*

In metastatic non-small-cell lung cancer (NSCLC), the role of local ablative radiation therapy (LAT) for oligoprogressive disease (OPD) on targeted therapy was first described by our group for EGFR- and ALK-positive cases. Here, we present an enlarged dataset, together with an extension of the analysis to other driver oncogenes.

*Methods*

A single-center, retrospective review of patients with metastatic NSCLC harboring *EGFR* mutations, *ALK* rearrangements, *ROS1* rearrangements and *BRAF* V600E mutations who had OPD on the respective tyrosine kinase inhibitor (TKI) and were treated with LAT was performed. OPD was defined as disease progression in ≤5 sites. Clinical characteristics, treatment regimens and treatment outcomes, including progression-free survival (PFS) and duration of treatment (DOT), were captured. PFS1 was defined as time from initiation of TKI-containing regimen to the first course of LAT for OPD. Subsequent PFS times (i.e., PFS2, PFS3, etc.) were defined as time from prior LAT to subsequent LAT or switch of systemic therapy, whichever occurred first. DOT was defined as time from initiation of TKI-containing regimen to switch of systemic therapy.

*Results*

A total of 75 patients were identified, including 46 with *EGFR* mutations, 19 with *ALK* rearrangements, 6 with *ROS1*rearrangements and 4 with *BRAF* V600E mutations. A total of 103 systemic therapies containing a TKI and 159 courses of LAT were recorded. Median line of therapy of TKI was 2 (range 1–6) and median LAT courses per systemic regimen was 1 (range 1–4). There were 50, 108 and 1 LAT treatment courses for central nervous system (CNS) OPD, extra-CNS OPD and both CNS and extra-CNS OPD, respectively. For extra-CNS LAT, the median number of sites of radiation per LAT was 1 (range 1–5). PFS1 was 9.1 months, and DOT was 17.3 months, implying a total extension of ~8 months on systemic therapy with at least one course of LAT. Median PFS per course of LAT was 4.8 months (95% CI 3.3–6.3 months). Subgroup analysis showed a similar benefit for each individual driver oncogene. Median PFS was longer for CNS LAT than extra-CNS LAT (6.2 months vs. 4.1 months, *p* = 0.024). For extra-CNS LAT, 12-month PFS was 12.2%, 6.1%, 0% and 0% for 1, 2, 3 and 5 sites, respectively; 24-month PFS was 8.7%, 0%, 0% and 0% for 1, 2, 3 and 5 sites, respectively.

*Conclusions*

LAT is effective in prolonging treatment duration for TKI-containing systemic therapy in oncogene-addicted NSCLC. The PFS extension is greater for CNS LAT compared to extra-CNS LAT. Prolonged PFS can be achieved in a subset of patients who have a single site of OPD.

## 5. Oral Presentation

**Nivolumab in Squamous Cell Carcinomas of the Head and Neck (SCCHN): A Real-World Outcome Study in Ontario, Canada**
Arman Zereshkian ^1^, Ruaa Shafi ^2^, Gregory Pond ^2^, Sebastien J. Hotte ^2^
^1^ Department of Medicine, McMaster University, Hamilton, ON, Canada
^2^ Department of Oncology, McMaster University, Hamilton, ON, Canada
* Correspondence: arman.zereshkian@medportal.ca

*Objective*

The CheckMate 141 trial led to the approval of nivolumab in platinum-resistant, metastatic squamous cell carcinomas of the head and neck (SCCHN). We evaluated outcomes of SCCHN patients in Ontario, Canada, treated with nivolumab since its funding through the New Drug Funding Program (NDFP) of Cancer Care Ontario (CCO).

*Methods*

A retrospective review using the provincial treatment registry (CCO NDFP) was undertaken. Patient characteristics, reason for treatment, treatment length, and date of death were collected. The Kaplan–Meier method was used to estimate overall survival and Cox regression to evaluate the prognostic effect of selected factors.

*Results*

A total of 134 patients with SCCHN received nivolumab. Median patient age was 63 years, with 80.6% being male. Nivolumab was used as second-line therapy after disease relapse within 6 months of curative-intent platinum chemotherapy (PC) in 39.6% (Indication 1—I1), used as second-line therapy post PC with non-curative intent in 42.6% (Indication 2—I2), and used as first-line therapy with non-curative intent due to contraindication for PC in 17.2% of patients (Indication 3—I3). Median overall survival (mOS) was 5.8 months, and one-year survival proportion was 28.4%. HPV status had no statistically significant impact on mOS (*p* = 0.12). Patients with a lower BSA (<1.81) had a mOS of 3.9 months versus 9.0 months for those with a higher BSA, HR = 0.12 (CI 0.04–0.39). Differences between indications were statistically significant (*p* < 0.001). Patients who received nivolumab for I1 had a mOS of 7.2 months versus 11.9 months for I3, HR = 1.73 (CI 0.94–3.16). Patients who received nivolumab for I2 had a mOS of 3.9 months versus 11.9 months for I3, HR = 3.27 (CI 1.80–5.94).

*Conclusions*

Real-world data in Ontario, Canada, demonstrated poorer mOS but a similar 1-year survival to the CheckMate 141 trial. HPV status had no impact on mOS. Patients who received nivolumab as first-line therapy in a non-curative setting appeared to have a greater mOS than those who received PC followed by nivolumab.

## 6. Oral Presentation

**Real-World Patient Eligibility for Lurbinectedin/Doxorubicin in Small-Cell Lung Cancer**
Rebekah Rittberg ^1^, Bonnie Leung ^1^, Zamzam Al-Hashami ^2^, Cheryl Ho ^1^
^1^ BC Cancer, Department of Medical Oncology, Vancouver, BC, Canada
^2^ Department of Medicine, College of Medicine & Health Sciences, Sultan Qaboos University, Muscat, Oman
* Correspondence: rebekah.rittberg@bccancer.bc.ca

*Background*

New drug approvals for small-cell lung cancer (SCLC) include first-line immune checkpoint inhibitors with chemotherapy and second-line lurbinectedin. In the ATLANTIS study, lurbinectedin/doxorubicin did not improve overall survival (OS); however, patients with a chemotherapy-free interval (CFI) of ≥180 days appeared to have an improved progression-free survival. The objective of this study was to identify the proportion of real-world SCLC patients who may be suitable for lurbinectedin/doxorubicin and to examine their characteristics.

*Methods*

A retrospective study of all SCLC cases referred to BC Cancer between 2012 and 2017 was conducted. BC Cancer is a provincial health care program that oversees cancer management for a population of 5.2 million. Patient demographics, staging, treatment, and survival information was collected. Baseline characteristics were compared using descriptive statistics including the X^2^ and Mann–Whitney U tests.

*Results*

A total of 1062 patients were identified. Baseline characteristics: median age 68, male 53%, never/former/current smoker 23 (2%)/381 (36%)/648 (61%), median pack years 50, limited stage 339 (32%), extensive stage 722 (68%). Systemic therapy was delivered to 821 (77%): first-line treatment was platinum doublet 748 (91%), single agent 65 (8%), CAV 4 (0.5%), other 4 (0.5%). A total of 173 patients received second-line treatment, and 60 received ≥3 lines. A total of 225 patients received first-line platinum doublet followed by ≥2 lines, and the CFI was ≥180 days for 104 patients. Characteristics by CFI < or ≥180 days: median age 66/63.5 (*p* = 0.97), male 45%/45% (*p* = 0.97), median pack years 40/44 (*p* = 0.14), limited stage 25%/50% (*p* < 0.001), OS from initiation of second line 4.2/8.6 months (*p* < 0.001).

*Conclusions*

In our real-world SCLC population, 10% of all diagnosed and 14% of platinum-treated patients were suitable for lurbinectedin/doxorubicin. The current, standard, second-line SCLC options continue to be the favored treatment for most patients. Further studies of lurbinectedin are required to identify the subpopulation who may most benefit.

## 7. Oral Presentation

**Survival Outcomes Associated with Chemotherapy-Induced Neutropenia in the Adjuvant Treatment of Colorectal Cancer with FOLFOX**
Sara Trincao-Batra ^1,2^, Rachel Goodwin ^1^, Horia Marginean ^1^, Jean Maroun ^1^, Michael Vickers ^1^, Timothy Asmis ^1^, Joanna Gotfrit ^1^
^1^ The Ottawa Hospital Cancer Center, University of Ottawa, Ottawa, ON, Canada
^2^ Memorial University of Newfoundland, St. John’s, NL, Canada
* Correspondence: strin009@uottawa.ca

*Background/Objective*

Patients undergoing adjuvant treatment with FOLFOX for colorectal cancer (CRC) are at risk of developing chemotherapy-induced neutropenia (CIN). We assessed survival outcomes in patients who develop CIN in this setting.

*Methods*

We performed a retrospective chart review of patients with CRC treated with FOLFOX at our institution in Canada from 2013 to 2015. Demographic, treatment, and outcome data were collected. CIN was defined as ANC < 1.5, and all episodes of neutropenia were assumed to be the result of chemotherapy. Median OS was calculated using Kaplan–Meier product limit estimates.

*Results*

A total of 302 patients were included (baseline demographics, Table 1). In the overall cohort, 174 (58%) patients had ≥1 episode of CIN. CIN occurred in 43% of those with low-risk stage III cancer (T1–3, N1) and 45% of those with high-risk stage III cancer (T4/N2). Among patients with ≥1 episode of CIN, 79 (45%) received subsequent granulocyte colony-stimulating factor (GCSF).

For patients with and without CIN, the median OS was not reached, HR 0.84 (95% CI 0.55–1.29, *p* = 0.43). The median OS for patients with CIN treated with and without GCSF was not reached, HR 1.02 (95% CI 0.57–1.82, *p* = 0.94). The 5-year survival rate for patients with and without CIN was 87% vs. 77%. The 10-year survival rate for patients with and without CIN was 70% vs. 64%. A trend toward improved survival in those with CIN remained when results were analyzed by cancer stage.

*Conclusions*

Patients with CIN had a trend toward improved survival compared to those who did not have CIN. There was no indication that GCSF in the setting of CIN impacted survival. Causes for the potentially protective effect of CIN in the setting of adjuvant CRC treatment require further elucidation.

## 8. Oral Presentation

**Karnofsky Performance Status (KPS) ≤60 is Strongly Associated with Shorter Brain-Specific Progression-Free Survival among Patients with Metastatic Breast Cancer with Brain Metastases**
Mark C. Freeman ^1^, Marguerite Ennis ^2^, Katarzyna J. Jerzak ^1,3,4^
^1^ Faculty of Medicine, University of Toronto, Toronto, ON, Canada
^2^ Applied Statistician, Markham, ON, Canada
^3^ Sunnybrook Research Institute, University of Toronto, Toronto, ON, Canada
^4^ Sunnybrook Odette Cancer Centre, Department of Medicine, University of Toronto, Toronto, ON, Canada
* Correspondence: markchaim.freeman@mail.utoronto.ca

*Objective*

To examine the association between Karnofsky performance status (KPS) and brain-specific progression-free survival (bsPFS) among patients with breast cancer brain metastases (BCBrM).

*Methods*

Using a previously compiled retrospective cohort of 683 patients who were treated for BCBrM with surgery and/or radiotherapy at the Sunnybrook Odette Cancer Centre from 2008 to 2018, electronic records were reviewed to impute KPS scores at the time of BCBrM diagnosis. Patients were then grouped into KPS ≤60 and KPS >60 cohorts. The dataset was analyzed to identify variables that were prognostic for bsPFS and/or overall survival (OS) under univariable and multivariable Cox proportional hazards models.

*Results*

The mean age of patients was 57 (range 24–93). Most patients (*n* = 622, 91%) had extracranial metastatic disease, and 174 (25%) had leptomeningeal disease. A total of 247 patients (36%) had hormone-receptor-positive/HER2-negative tumors, 189 (28%) had HER2-positive disease, and 153 (22%) had triple-negative breast cancer. Of the 331 patients (48%) who could be assigned a KPS cohort, 102 (31%) had KPS ≤60. Most patients were treated with whole-brain radiotherapy (*n* = 498, 73%) and/or stereotactic radiosurgery (SRS) (*n* = 128, 19%). Median bsPFS was 9 months (95% CI 8–10 months), and median OS was not reached. In univariable analyses, KPS ≤60, presence of leptomeningeal disease, neurological symptoms, ≥2 brain metastases, and not undergoing SRS were factors associated with shorter bsPFS. In a multivariable analysis, KPS ≤ 60 was the only statistically significant determinant of bsPFS (HR 1.86, 95% CI 1.20–2.88). Although survival data were limited, KPS ≤60 was associated with shorter OS in both univariable (HR 3.12, 95% CI 1.85–5.26) and multivariable (HR 2.95, 95% CI 1.55–5.58) analyses.

*Conclusions*

Patients with BCBrM who have a KPS ≤60 have significantly shorter bsPFS and OS than those with KPS >60. KPS should be documented routinely at the time of diagnosis of brain metastases to improve prognostication.

## 9. Oral Presentation

**Role of Serum Tumor Biomarkers in the Diagnosis and Objective Clinical Assessment of Non-Small-Cell Lung Cancer**
Scott W Strum ^1,2^, Mark Vincent ^1,2^, Meghan Gipson ^3^, Eric McArthur ^2^, Dan Breadner ^1,2^
^1^ Department of Oncology, Schulich School of Medicine and Dentistry, London, ON N6A 5W9, Canada
^2^ London Regional Cancer Program at London Health Sciences Centre, London, ON N6A 5W9, Canada
^3^ Department of Medicine, Royal College of Surgeons in Ireland, Dublin D02 YN77, Ireland
* Correspondence: sstrum@uwo.ca

*Objective*

The purpose of this study was to assess whether three commonly available, low-cost serum biomarkers (CEA, CA19-9, and CA-125) correlated with radiographic disease response/progression during or after the treatment of non-small-cell lung cancer (NSCLC), regardless of stage, histologic classification, or type of systemic therapy.

*Methods*

A retrospective, single-center review of NSCLC patients at the London Regional Cancer Program between 1 January 2016 and 1 August 2020. This was a planned futility interim analysis to assess the first 50 patients with imaging and tumor markers at baseline and at least one follow-up time-point. Disease response/progression was assessed radiographically using RECIST 1.1 criteria. Statistical analyses were completed using paired nonparametric tests (Wilcoxon signed-rank test).

*Results*

A total of 50 patients were analyzed. At baseline imaging, an elevated CEA was seen in 54% of patients, elevated CA19-9 in 32% of patients, and elevated CA-125 in 42% of patients. The median fold change in the tumor marker from its nadir to its level at progression (*n* = 32) was 2.16 (1.18, 3.23; *p* <0.001) for CEA, 1.46 (1.19, 2.20; *p* <0.001) for CA19-9, and 1.65 (1.10, 3.31; *p* < 0.001) for CA-125. The median fold change in the tumor marker from baseline to its level at first response was CEA 0.82 (0.35, 0.95; *p* = 0.025), CA19-9 0.97 (0.56, 1.40; *p* = 0.44), and CA-125 0.84 (0.17, 1.29; *p* = 0.28). The best radiographic response was progression in 18% of patients, partial response in 44%, and stable disease in 38%.

*Conclusions*

This planned futility analysis after the first 50 patients revealed that CEA, CA19-9, and CA-125 all demonstrate statistically significant associations with NSCLC disease progression. CEA showed a statistically significant correlation with treatment response. These results justify further data collection to allow for a more robust analysis, including subgroup analysis based on stage, histology, and type of systemic therapy.

## 10. Oral Presentation

**Real-World Analysis of Outcomes of Patients Receiving Bevacizumab for Recurrent Glioblastoma in British Columbia**
Manik Chahal ^1^, Brian Thiessen ^1^
^1^ BC Cancer, Vancouver Centre, Department of Medical Oncology, Vancouver, BC, Canada
* Correspondence: manik.chahal@phsa.ca

*Background*

Bevacizumab (Bev) has been publicly funded in British Columbia (BC) since 2011 for treatment of recurrent glioblastoma (rGBM). We performed a retrospective outcomes assessment of patients with rGBM treated with Bev.

*Methods*

Patients with rGBM treated at BC Cancer centers with Bev between January 2011 and December 2017 were reviewed. Patient demographics, tumor characteristics, treatment regimens, and dates of radiographic progression and death were collected. The Kaplan–Meier method was used to assess survival, and comparisons were made using the log-rank test.

*Results*

A total of 182 patients were reviewed. There were 180 reported deaths with a median PFS of 3 months (CI_95_ = 2.5–3.5) and an OS of 7 months (CI_95_ = 6.2–7.8) from Bev initiation. A higher corticosteroid dose prior to Bev initiation was associated with worse OS in univariate and multivariate analyses (*p* = 0.40 and 0.01, respectively). Of the patients on corticosteroids, 67% reduced their dose shortly after Bev initiation. Most patients (68%) were treated with multiple lines of therapy prior to Bev, with a median time from chemoradiation to Bev initiation of 8 months (range 1–67). Patients that started on Bev < 6 months from chemoradiation (prior to adjuvant temozolomide completion) had improved PFS (*p* = 0.02) and OS compared to those who started Bev later (*p* = 0.05), but there was no association between extent of treatment prior to Bev and outcomes (*p* = 0.09). The addition of chemotherapy to Bev did not improve survival over Bev monotherapy (*p* = 0.10).

*Conclusions*

Bev combinations with chemotherapy did not confer a survival advantage over Bev monotherapy. Furthermore, our results showed that patients receiving Bev before completion of adjuvant chemotherapy have better outcomes, suggesting pseudoprogression prompts therapeutic switch. Further research is required to optimize patient selection for and administration of Bev. Additional analysis of rGBM patients prescribed Bev up to 2020 in BC is currently underway.

## 11. Oral Presentation

**Applying the eHEALS Scale to Assess the Degree of Electronic Literacy among Geriatric Patients Attending a Skin Cancer Clinic at Princess Margaret Cancer Centre**
Mauricio F. S. A. Ribeiro ^1^, Thiago P. Muniz ^1^, Nancy Gregorio ^1^, Diana Gray ^1^, Raviya Singh ^1^, Samuel Saibil ^1^, Marcus Butler ^1^
^1^ Division of Medical Oncology and Hematology, Princess Margaret Cancer Centre, Toronto, ON, Canada
* Correspondence: mauricio.ribeiro@uhn.ca

*Objectives*

To assess the perceived importance and level of electronic health literacy (EHL) of the geriatric population attending a skin cancer clinic.

*Methods*

We performed convenience sampling of ^3^70-year-old skin cancer patients attending regular visits at the Princess Margaret Cancer Centre since December 2021. Their ability to handle technology was measured through the validated eHEALS tool, which provides overall scores between 8–40, with higher values indicating more technology skills to obtain and apply health information. Correlation between age and eHEALS scores was assessed by the Spearman test (two-tailed *p* < 0.05 considered statistically significant). Information regarding steroid use, active treatment, and interruption or discontinuation due to toxicities was also collected.

*Results and Conclusions*

In 50 patients assessed, the median age was 78 (70–87), 34 (68%) were male, 47 (94%) had ECOG-PS 0–1, and 31 (62%) had cutaneous melanomas. Thirty-five (70%) were on active treatment: 14 with anti-PD1 and anti-CTLA4, 16 with anti-PD1 in monotherapy, and 5 with other regimens. Nineteen (38%) patients interrupted or discontinued treatment due to toxicities, and 13 (26%) were on steroids. Twenty (40%) patients did not consider it important to have access to health resources on the internet, and 22 (44%) did not use the internet to make health decisions, relying totally on close relatives and healthcare providers. The mean eHEALS score was 25.24 (SD 9.05), and no correlation between increased age and low eHEALS was identified (s = −0.15, 95% CI -0.427–0.146, *p* = 0.28). Moreover, 11 (22%) individuals did not agree with any of the eHEALS statements. These results suggest that low EHL is a reality among patients ^3^70-year-old and could pose additional hurdles in handling electronic tools otherwise developed to facilitate communication. Adopting eHEALS in our practice may be a time-saving strategy to identify those necessitating more assistance as we move towards broader use of digital tools to improve care.

## 12. Oral Presentation

**A Residency Training Program Analysis of Paclitaxel Infusion Reactions: Are We Over-ReACTInG?**
Jamie Feng^1^, Alghawas, M. ^1^, Cheng, P. ^1^, Jackson, E. ^1^, Jones, L. ^1^, Li, K. ^1^, Poon, V. ^1^, Wells, C. ^1^, Leung, B. ^2^ and Simmons, C. ^1,2^
^1^ Medical Oncology Residency Training Program, University of British Columbia, Vancouver, BC, Canada
^2^ BC Cancer Vancouver Centre, Vancouver, BC, Canada
* Correspondence: jamie.feng@bccancer.bc.ca

*Background*

Medical oncology trainees at BC Cancer, Vancouver, respond to acute hypersensitivities during chemotherapy. Anecdotally, an increasing number of drug reaction pager (DRP) calls that require in-person assessment for drugs such as paclitaxel has been seen. This has negative impacts on direct patient care, and, indirectly, patient flow. Our quality improvement project “REsident-led Assessment of ChemoTherapy Infusion reaction Guidelines” (ReACTInG) aims to decrease DRP calls.

*Methods*

We propose a series of Plan–Do–Study–Act (PDSA) cycles to clarify the need for in-person assessment of DRPs and decrease the number of paclitaxel infusion reactions. Our multifaceted approach includes nursing education (slated to begin March 2022) and a prospective cohort study for breast and gynecological cancer patients receiving paclitaxel using standard versus high(er)-dose dexamethasone prophylaxis. This is planned to start in September 2022, pending ethics approval.

*Results*

We report the baseline frequency and nature of DRP calls at BC Cancer, Vancouver, from 30 August 2021 to 22 October 2021. Over the course of 8 weeks, 64 calls were recorded. On independent review, 49 (76.5%) were deemed appropriate (grade 2 or above), while 14 were grade 1 as per BC Cancer guidelines. Paclitaxel accounted for nearly half of the calls (48.4%; 31/64). An estimated total provider time of 1269 min was needed to respond to these calls, averaging 19 min per call. During a four-week block, residents in the medical oncology training program were missing, on average, two half-day clinics to manage these calls.

*Conclusions*

Our findings support that the largest proportion of DRP calls are due to paclitaxel hypersensitivity and will be the baseline for our cohort study. Through this resident-led initiative we endeavor to limit the number of (low-grade) DRP calls, specifically paclitaxel infusion reactions, to help decrease patient distress and personnel/resource costs.

**Award Recipient**

## 13. Poster Presentation

**Comparison of Two-Weekly Versus Four-Weekly Durvalumab Consolidation for Advanced NSCLC Treated with Chemoradiotherapy**
Marie-Hélène Denault ^1,2^, Shelley Kuang ^1^, Aria Shokoohi ^1^, Bonnie Leung ^1^, Mitchell Liu ^1^, Eric Berthelet ^1^, Janessa Laskin ^1^, Sophie Sun ^1^, Tina Zhang ^1^, Barbara Melosky ^1^, Cheryl Ho ^1^
^1^ BC Cancer Agency-Department of Medical Oncology, University of British Columbia, Vancouver, BC, Canada
^2^ Centre de recherche de l’Institut universitaire de cardiologie et de pneumologie de Québec-Département de pneumologie et de chirurgie thoracique, Université Laval, Québec, QC, Canada.
* Correspondence: mariehelene.denault@bccancer.bc.ca

*Introduction*

Durvalumab 10 mg/kg every two weeks for one year after chemoradiation has improved overall survival (OS) in unresectable stage III non-small-cell lung cancer (NSCLC) [1,2]. Subsequently, a 20 mg/kg four-weekly regimen was approved. The study goal was to compare the efficacy and toxicity of the two regimens.

*Methods*

We reviewed the medical records of all NSCLC patients treated with curative-intent chemoradiation followed by durvalumab from 1 March 2018 to 31 December 2020 at BC Cancer, British Columbia, Canada. The four-weekly dose was introduced in April 2020. The information on the durvalumab dosing schedule, toxicity, progression pattern and survival data was collected. Patients were divided according to the dosing schedule that was used for the majority of treatment. Comparisons were made using chi-square and independent t tests. Kaplan–Meier curves and a log-rank test were used to analyze overall survival.

*Results*

A total of 152 patients were included in the two-weekly group and 53 patients in the four-weekly group. Median follow-up was 19.7 months and 12.0 months, respectively. Most patients received carboplatin, and over 90% completed both chemotherapy (≥2 cycles) and radiation (≥60 Gy). Median OS was not reached, but 12-month survival rates were 88.4% versus 85.2% (*p* = 0.55). Toxicity profiles were similar in terms of sites and severity.

*Conclusions*

There was no significant difference in efficacy or toxicity between the two-weekly and four-weekly durvalumab in this cohort of advanced NSCLC patients previously treated with curative-intent chemoradiation.

References

1. Antonia, S.J.; Villegas, A.; Daniel, D.; Vicente, D.; Murakami, S.; Hui, R.; Yokoi, T.; Chiappori, A.; Lee, K.H.; de Wit, M.; et al. Durvalumab after Chemoradiotherapy in Stage III Non–Small-Cell Lung Cancer. *N. Engl. J. Med.* **2017**, *377*, 1919–1929.
2. Spigel, D.R. Five-Year Survival Outcomes with Durvalumab after Chemoradiotherapy in Unresectable Stage III NSCLC: An Update from the PACIFIC Trial in 2021 ASCO Annual Meeting. *J. Clin. Oncol.* **2021**, *40*, 1301.

## 14. Poster Presentation

**Measles, Mumps, and Rubella (MMR) Reactivity and Vaccination Eligibility in Autologous Hematopoietic Stem Cell Transplant (HSCT) Recipients**
Olivia Owen ^1^, Gopika Punchhi ^1^, Hammad Saif ^1^, Aman Sehmbi ^1^, Sharon Pritchard ^1^, Kayla Negus ^1^, Caroline Hamm ^1,2^
^1 ^ Schulich School of Medicine & Dentistry
^2 ^ Windsor Regional Cancer Centre
* Correspondence: oowen2024@meds.uwo.ca

*Objective*

We aimed to assess measles, mumps, and rubella (MMR) reactivity in autologous hematopoietic stem cell transplant (HSCT) recipients and eligibility for live vaccine administration at two years post HSCT in recipients who required MMR revaccination to inform current guidelines on post-HSCT vaccination.

*Methods*

We completed a retrospective chart review of 69 autologous HSCT recipients at the Windsor Regional Cancer Centre transplanted between June 2016 and January 2020 to assess post-transplant MMR reactivity. In those without reactivity, we assessed eligibility for revaccination based on administration of contraindicated medications at two years post HSCT, as stated in the most recent Canadian Immunization Guidelines.

*Results*

The most common indication for autologous HSCT was multiple myeloma, with 68% of patients having this diagnosis. Of those with post-HSCT MMR reactivity assessments (*n* = 58), 55%, 72%, and 48% had non-reactive or indeterminate status to measles, mumps, or rubella, respectively. While 67% were reactive to at least one of the three, only 10% of these patients were fully reactive to MMR. Of living patients who required revaccination 2 years post HSCT (*n* = 47), 47% were on a contraindicated medication with the most common medications being dexamethasone or prednisone (23%), bortezomib (8.5%), and carfilzomib (8.5%).

*Conclusions*

The majority of autologous HSCT recipients lack MMR reactivity post HSCT and require revaccination; however, many recipients are not eligible for revaccination based on current guidelines. The safety and efficacy of MMR revaccination with contraindicated medications should be evaluated to enable a greater proportion of susceptible patients to be revaccinated. 

## 15. Poster Presentation

**Improving First-Line Systemic Therapies: The Impact of Bevacizumab on Survival Outcomes of High-Risk Ovarian Cancer Patients**
Raheesa Jina ^1^, Longlong Huang ^2^, Shaun Sun ^2^, Jenny Ko ^3^
^1^ Faculty of Medicine, University of British Columbia, Vancouver, BC, Canada
^2^ Department of Mathematics and Statistics, University of the Fraser Valley, Abbotsford, BC, Canada
^3^ Department of Medical Oncology, BC Cancer-Abbotsford, Abbotsford, BC, Canada
* Correspondence: raheesa@student.ubc.ca

*Objective*

There is limited real-world data to address the efficacy of upfront bevacizumab, an anti-VEGF monoclonal antibody, on survival in epithelial ovarian cancer (EOC) patients. This study compares survival outcomes in advanced, high-risk EOC patients across British Columbia who received standard treatment with or without first-line bevacizumab.

*Methods*

All consecutive patients with inoperable or sub-optimally debulked stage III or stage IV EOC (high-risk by ICON7 criteria) diagnosed between 1 January 2018 and 31 December 2020 were identified through the provincial cancer health record. Survival outcomes were stratified based on receipt of upfront bevacizumab, FIGO stage, performance of surgery, disease relapse, and number of chemotherapy cycles. Univariate analysis (log-rank tests and Kaplan–Meier curves) and multivariate analysis were performed.

*Results*

A total of 166 patients with high-risk disease were identified; the median age at time of diagnosis was 67 years, with 71% having stage IV disease. With regards to treatment, 11% had upfront bevacizumab (6% induction therapy, 42% maintenance therapy, 67% both therapies), and the remaining 89% received standard chemotherapy alone. Univariate analysis showed that patients receiving first-line bevacizumab had significantly longer relapse-free survival (RFS) and overall survival (OS) compared to controls (*p* = 0.0065 and *p* = 0.034, respectively). With multivariate analysis, a statistically significant improvement in RFS (HR = 0.168, *p* = 0.016) and an improvement in OS that was not statistical significance (HR 0.232, *p* = 0.052) were noted.

*Conclusions*

Our study confirms that use of first-line bevacizumab is associated with better RFS and a trend towards better OS in high-risk, advanced-stage EOC patients.

## 16. Poster Presentation

**The Use of Salvage Chemotherapy for Patients with Relapsed Testicular Germ Cell Tumor (GCT) in Canada: A National Survey Study**
ESMAIL AL-EZZI ^1^, Carlos Stecca ^1^, Rob Hamilton ^2^, Michael Crump ^1^, John Kuruvilla ^1^, Lori Wood ^3^, Lucia Nappi ^4^, Christian Kollmannsberger ^4^, Scott North ^5^, Eric Winquist ^6^, Denis Soulieres ^7^, Sebastien Hotte ^8^, Srikala Sridhar ^1^, Di Maria Jiang ^1^
^1^ Division of Medical Oncology and Hematology, Princess Margaret Cancer Centre, University Health Network, University of Toronto, Toronto, ON, Canada
^2^ Division of Urology, Department of Surgery, Princess Margaret Cancer Centre, University Health Network, University of Toronto, Toronto, ON, Canada
^3^ Division of Medical Oncology, Queen Elizabeth II Health Sciences Centre, Dalhousie University, Halifax, NS, Canada
^4^ Department of Medicine, British Columbia Cancer Agency, University of British Columbia, Vancouver, BC, Canada
^5^ Division of Medical Oncology, Cross Cancer Institute, University of Alberta, Edmonton, AB, Canada
^6^ Department of Oncology, London Health Sciences Centre, Western University, London, ON, Canada
^7^ Département Hématologie-oncologie, Centre Hospitalier de l′Université de Montréal, Montréal, QC, Canada,
^8^ Department of Oncology, Juravinski Cancer Centre, McMaster University, Hamilton, ON, Canada
* Correspondence: esmail.al-ezzie@uhn.ca

*Background*

Although metastatic GCT is highly curable, 10% of patients relapse after initial cisplatin-based chemotherapy and have a poorer prognosis. Salvage chemotherapy options include conventional-dose chemotherapy (CDCT) and high-dose chemotherapy (HDCT). However, definitive comparative data remain lacking. We aimed to characterize the contemporary practice patterns of salvage chemotherapy across Canada.

*Methods*

We conducted a 30-question online survey in August 2021 on medical oncologists (MO) and hematological oncologists (HO) with experience in treating GCT, assessing treatment availability, patient selection, and management strategies used for relapsed GCT patients.

*Results*

Respondents included: 25 staff MO, 3 HO, and 2 who were both; from British Columbia, Alberta, Manitoba, Ontario, Quebec, New Brunswick, Nova Scotia, and PEI; 86% were from academic centers. Reported case volumes for salvage chemotherapy were <1 (18%), 1 (21%), 1–5 (39%), and 6–10 cases/year (21%). No active clinical trials were available at the time of the survey. The most common CDCT regimens used were TIP (64%) and VIP (25%). HDCT was available for 70% and used as first- (67%; range 0–100), second- (33%; 0–100) or third-line/beyond (4%; 0–20) salvage therapy. Only some used the IPFSG risk classification for treatment selection (Figure 1). Assuming tolerability and feasibility, only one respondent indicated a clinical scenario precluding HDCT (“rising markers during platinum chemotherapy for mediastinal nonseminoma”). The HDCT regimen used included carboplatin and etoposide (two cycles 76%; three cycles 6%) and the TICE protocol (two centers). “Bridging” CDCT was needed by 63% while waiting to access HDCT. Post-HDCT treatments considered included surgical resection for residual disease (*n* = 13), maintenance etoposide (*n* = 1), and surveillance only (*n* = 1).

*Conclusions*

HDCT is the most commonly used GCT salvage strategy in Canada. Significant differences exist in the treatment availability, selection, and delivery of HDCT, highlighting the need for standardization of care for patients with relapsed testicular GCT requiring salvage chemotherapy. 

## 17. Poster Presentation

**In-House Molecular Biomarker Testing and Time to Treatment Decision in Advanced Non-Small Cell Lung Cancer**
Grace K. Grafham ^1,2^, Kenneth J. Craddock ^2^, Weei-Yuarn Huang ^2^, Alexander V. Louie ^3^, David M. Hwang ^2^, Ambica Parmar ^1^
^1 ^ Division of Medical Oncology & Hematology, Department of Medicine, Sunnybrook Health Sciences Centre, Toronto, ON, Canada
^2 ^ Department of Laboratory Medicine & Molecular Diagnostics, Sunnybrook Health Sciences Centre, Toronto, ON, Canada
^3 ^ Department of Radiation Oncology, Sunnybrook Health Sciences Centre, Toronto, ON, Canada
* Correspondence: grace.grafham@mail.utoronto.ca

*Objective*

To evaluate differences in time to treatment decision (TTD) following implementation of in-house molecular biomarker testing as compared to send-out testing for advanced non-small-cell lung cancer (NSCLC) at our institution.

*Methods*

We retrospectively reviewed patients with newly diagnosed stage IV NSCLC treated at Sunnybrook Health Sciences Centre (Toronto, Ontario) between March 2017 and March 2021. Data were compared during the send-out (March 2017–May 2019) and in-house (July 2019–March 2021) testing periods. Polymerase-chain-reaction-based assays were used for *EGFR* analysis, and immunohistochemical assays were used for assessment of *ALK* and PD-L1. TTD was defined as the interval between the pathological diagnosis of NSCLC and the decision for treatment with systemic therapy. We performed a detailed workflow analysis to provide insight on the pre-testing, testing, and post-testing intervals that constituted the total TTD.

*Results*

A total of 165 patients were included (*n* = 92 for send-out testing, *n* = 73 for in-house testing). Of the patients with in-house testing, 88% had results available at their initial oncology consultation compared to only 52% of patients with send-out testing (*p* < 0.0001). Median TTD was significantly faster with in-house testing (14 days (interquartile range [IQR], 8–25 days) vs. 25 days (IQR, 21–38 days), *p* < 0.0001). This improvement was largely driven by decreased internal handling/specimen transit times (2 days (IQR, 1–2 days) vs. 4 days (IQR, 3–6 days), *p* < 0.0001) and laboratory turnaround times (TAT, 4 days (IQR, 2–7 days) vs. 12 days (IQR, 9–15 days), *p* < 0.0001), with 96% of in-house cases meeting the international recommended guideline of a ≤14-day TAT (vs. 74%, *p* < 0.001). In-house testing had no impact on the post-testing interval (9 days (IQR, 4–16 days) vs. 9 days (IQR, 5–17 days), *p* = 0.86).

*Conclusions*

In-house testing of molecular biomarkers for advanced NSCLC was successfully implemented at our institution and was associated with a significant reduction in TTD.

## 18. Poster Presentation

**HPV-Related Squamous Cell Carcinoma of the Larynx in Two Young Female Patients**
Zahra Taboun ^1^, Peter Zeng ^1^, Jasna Deluce ^1,2^, Kevin Fung ^3^, Anthony Nichols ^3^, John Barrett ^4^, Lama Elkadri ^4^, David Palma ^5^, Paul Stewart ^2^, Matthew Cecchini ^6^, Eric Winquist ^1,2^
^1 ^ Schulich School of Medicine & Dentistry, Western University, London, ON, Canada
^2 ^ Division of Medical Oncology, Department of Oncology, Western University and London Health Sciences Centre, London, ON, Canada
^3 ^ Department of Otolaryngology/Head and Neck Surgery, Western University and London Health Sciences Centre, London, ON, Canada
^4 ^ Lawson Health Research Institute, London Health Sciences Centre, London, ON, Canada
^5 ^ Division of Radiation Oncology, Department of Oncology, Western University and London Health Sciences Centre, London, ON, Canada
^6 ^ Department of Pathology and Laboratory Medicine, Western University and London Health Sciences Centre, London, ON, Canada
* Correspondence: ztaboun@uwo.ca

*Objective*

The goal of this project was to investigate the potential etiology, treatment, and course of disease for two females under 30 years of age with squamous cell carcinoma of the larynx and no/light smoking history.

*Methods*

With their consent, the medical records of the two patients were reviewed. Genomic DNA was extracted from FFPE pathology slides and whole-exome sequencing performed. Amplified products were Sanger sequenced and aligned to reference HPV sequences to confirm their identity. Mutations were identified using MuTect2 (v4.17.0) and annotated using SnpEff. HPViewer was used to identify the specific HPV genotype.

*Results*

Case 1: An 18-year-old female presented with a six-year history of hoarseness and one-year history of sore throat, difficulty swallowing, and weight loss. She had no comorbid medical conditions and was a non-drinker and a non-smoker. She was diagnosed with T4N2b squamous cell carcinoma of the glottic larynx and, subsequently, underwent a total laryngectomy, bilateral neck dissection, and total thyroidectomy, followed by chemoradiation. She has remained disease free for 15 years. Molecular testing identified HPV45, and exome sequencing identified 312 mutations including FAT1 and FAT2.

Case 2: A 24-year-old female presented with a one-year history of a hoarse voice. There was a 10-pack-year smoking history. She was diagnosed with cT3N0 squamous cell carcinoma of the left vocal cord and treated with chemoradiation therapy. There is no evidence of recurrence 19 months post treatment. Molecular testing identified HPV31, and exome sequencing identified 95 mutations including NOTCH1, MAPK1 (ERK2), and HIST1H2AK.

*Conclusions*

Laryngeal carcinoma is rare in young individuals. Our data suggest that these cancers may be quite different biologically from smoking-related cancers in older adults. These data provide a rationale for further investigation into the etiology, prevention, and treatment of these cancers. Specifically, HPV vaccination and organ preservation strategies may be particularly relevant and effective.

## 19. Poster Presentation

**Clinical Experience of Patients Referred to a Multidisciplinary Cardio-Oncology Clinic in Northwestern Ontario: An Observational Cohort Study**
Andres, G.G.G. ^1,2^, Smylie, P. ^2^, Svyst, H. ^3^, Docherty, A. ^1,4^, Roberts, K. ^1,4^, Melenchuk, K. ^1,4^, Alaref, A.A. ^1,2^, Rohani, A. ^1,2^, Laferriere, N. ^1,4^ and Aseyev, O.I. ^1,4^
^1^ Thunder Bay Regional Health Sciences Centre, Thunder Bay, ON, Canada
^2^ NOSM University, Thunder Bay, ON, Canada
^3^ Carleton University, Ottawa, ON, Canada
^4^ Regional Cancer Care Northwest, Thunder Bay, ON, Canada
* Correspondence: agriborioguzman@nosm.ca

*Objective*

This study aims to report the experience of a cardio-oncology clinic recently established at the Thunder Bay Regional Health Sciences Centre (TBRHSC) to contribute to the literature on the emerging field of cardio-oncology and compare it with experiences at similar centers.

*Methods*

This retrospective observational study included cancer patients referred to the TBRHSC Cardio-Oncology Clinic (COC). Patient demographics, cancer type, the reason for referral, cardiovascular risk factors, cancer and cardiac therapies, and clinical outcomes were collected. Between January 2018 and June 2019, 124 patients (71 women, 57%; 53 men, 43%) were referred to the COC. The median age of the patients at cancer diagnosis was 62 years (range: 23–83 years).

*Results*

The most common types of primary malignancy were hematologic (38%) and breast (29%). The most frequent reasons for referral were clinically suspected cardiotoxicity (69%), history of acute coronary syndrome (32%), abnormal blood pressure (26%), and heart failure (26%). Treatment with cardiac medication was given in 58 patients (47%), with 24 (19%) receiving a beta blocker, 7 (6%) receiving an angiotensin-converting enzyme (ACEi), and 12 (10%) receiving an ACEi and a beta blocker. During or before the study period, 29 patients (23%) were able to complete their prescribed cancer therapy with COC co-management, with 74 patients (60%) having ongoing treatment and 21 patients (17%) ceasing therapy. Most of the 124 patients (*n* = 104, 84%) were alive at the time of the last data collection.

*Conclusions*

This cohort study is the first to report and compare the characteristics and clinical outcomes of patients referred to a COC in Northwestern Ontario and one of the few that includes patients receiving radiotherapy. Collaboration between oncologists and cardiologists resulted in most patients completing cancer therapy. Analyzing referral patterns, management plans, and patient outcomes will help improve cancer and cardiac outcomes for oncology patients.

**Keywords:** cardio-oncology; cardiotoxicity; targeted therapy; LVEF; cardiac outcomes

## 20. Poster Presentation

**Staff Experience with Remote Work in A Comprehensive Cancer Center during the COVID-19 Pandemic**
Christopher McChesney ^1,2,3^, Melanie Powis ^1^, Lyndon Morley ^1,4^, Saidah Hack ^1^, Monika Krzyzanowska ^1,2,3^
^1^ Cancer Quality Lab (CQuaL), Princess Margaret Cancer Centre, University Health Network, Toronto, ON, Canada
^2^ Department of Medicine, Faculty of Medicine, University of Toronto, Toronto, ON, Canada
^3^ Institute of Health Policy, Management and Evaluation, University of Toronto, Toronto, ON, Canada
^4^ Radiation Medicine Program, Princess Margaret Cancer Centre, University Health Network, Toronto, ON, Canada
* Correspondence: chris.mcchesney@utoronto.ca

*Background*

The COVID-19 pandemic led to the rapid implementation of remote work, but few studies have evaluated how this impacted staff and hospital operations. Utilizing a cross-sectional survey, we evaluated the impact of remote work on clinical staff at the Princess Margaret Cancer Centre.

*Methods*

“Remote work” was defined as any work (tasks, projects, healthcare delivery) performed from home. A Qualtrics survey was disseminated via email three times from June 2021 to August 2021 to 1168 physicians, nurses, and allied health and administrative staff normally involved in patient care. The survey evaluated staff perceptions of productivity and efficiency, patient safety/quality, personal experience, and physical workspace. Results were summarized using descriptive statistics. Free-text responses were categorized into facilitators and barriers using qualitative descriptive analysis.

*Results*

Most respondents (*n* = 333; response rate: 28.5%) were female (61.3%) and physicians (23.1%). Very few respondents (1.5%) worked remotely more than half the time prior to COVID; this increased to 66.6% since COVID. Most of respondents reported that working remotely positively impacted productivity (61.8%) and efficiency (57.6%) and expressed interest in continuing (79.0%). While most respondents agreed with the switch to remote work (89.2%), few were provided with the needed equipment (14.1%). In general, respondents indicated that the safety and quality of care delivered remotely were equivalent to that delivered on site, with 13.8% and 18.6% perceiving a negative impact on safety and quality, respectively. Facilitators of remote work included improved efficiency, less commuting, and improved work–life balance. Barriers included a lack of clear expectations for roles, issues accessing applications, and out-of-pocket expenses.

*Conclusions*

Our findings indicate that working remotely is acceptable to clinical staff, and most staff perceive the quality and safety of care to be adequate. However, policies standardizing roles and expectations across clinical services, and provision of additional training and equipment are needed to successfully sustain remote work in healthcare moving forward.

## 21. Poster Presentation

**Local Use of Nivolumab in Esophageal and Gastroesophageal Junction Cancers: Real-World Evidence from Nova Scotia**
Margaret Sheridan ^1^, Stephanie Snow ^1^
^1^ Dalhousie University
* Correspondence: margaret.sheridan@dal.ca

*Introduction*

Post-operative immunotherapy (POI) with nivolumab has become the standard of care for eligible patients with esophageal and gastroesophageal junction cancers (EGEJC) who had pre-operative chemoradiation (POCRT) and definitive surgery. However, not all patients who receive POCRT are eligible for POI.

*Objective*

The primary objective was to determine the proportion of patients who receive POCRT that are eligible for POI in a real-world population. Further, we aimed to identify the specific barriers to POI to determine factors which could be strategically addressed to maximize access to curative-intent treatment.

*Methods*

All patients residing in mainland Nova Scotia who received POCRT between July 2016 and April 2019 were analyzed. Disease, patient, and treatment characteristics were collected via chart review. The relative proportion of patients who (1) had definitive surgery, (2) were eligible to receive POI, and (3) would have accepted POI were collected. The specific reasons for non-eligibility or refusal of POI were captured.

*Results*

A total of 65 patients received POCRT during the study period. Of those, 44 went on to have definitive surgery. All 44 of these patients were then screened for eligibility to be enrolled in a trial of POI. A total of 23 were eligible, but 12 declined trial enrolment. A total of 21 were ineligible, with the most common reason being a pathological complete response (*n* = 11). Of the 21 patients who did not undergo surgery, 12 were found to have new metastatic disease on pre-operative restaging, and nine were felt to be too frail.

*Conclusions*

Only 35% of patients receiving POCRT were eligible for POI. These are important real-world data when planning allocation of resources and adapting health care budgets for novel treatments and indications.

## 22. Poster Presentation

**Real-World Eligibility of Patients with Metastatic Breast Cancer (MBC) for Clinical Trials on the Basis of “Stable” Brain Metastases (BrM)**
Isabella Kojundzic ^1^, Rania Chehade ^2,3^, Mark C. Freeman ^2,3^, Katarzyna J. Jerzak ^1,2,3^
^1 ^ Sunnybrook Research Institute, University of Toronto, Toronto, ON, Canada
^2 ^ Faculty of Medicine, University of Toronto, Toronto, ON, Canada
^3 ^ Sunnybrook Odette Cancer Centre, Department of Medicine, University of Toronto, Toronto, ON, Canada
* Correspondence: isabella.kojundzic@mail.utoronto.ca

*Objective*

Determine the proportion of patients with MBC who meet common clinical trial criteria for “stable” BrM.

*Methods*

Using a retrospective cohort of 751 patients treated with local therapy for breast cancer BrM between 2008 and 2018 at the Sunnybrook Odette Cancer Centre, we evaluated the proportion who meet common stipulations for stable BrM in clinical trials. Given that patients are typically evaluated for clinical trials on the basis of systemic disease progression (with or without intracranial progression), we identified a subset of patients whose systemic disease progressed within 4 weeks before or after local therapy for BrM with surgery and/or radiotherapy.

*Results*

Of 751 patients, 413 (55%) had extracranial disease progression, and 300 (40%) had stable extracranial disease at the time of treatment of BrM; the status of extracranial disease was unknown among 38 (5%) of patients. Among 413 patients with concurrent intra- and extracranial disease progression, the median age at BrM diagnosis was 57 (range 24–93); 45.7% (*n* = 189/413) had a Karnofsky performance status (KPS) ≥ 60, and 16.7% (*n* = 69/413) received stereotactic radiosurgery (SRS) for BrM. A total of 22.8% (94/413) had triple-negative breast cancer, 22.5% (93/413) had HER2+, 39.7% (164/413) had hormone receptor (HR)+/HER2− disease, and 15% (62/413) had unknown receptor status. The proportion of patients who failed to meet common criteria for “stable” BrM varied by the definition used to define stability. A total of 312 (72.5%) of patients had symptomatic BrM, 210 (50.8%) required local therapy within 4 weeks of extracranial progression, and 1 (0.2%) had “active”/progressive BrM that was not amenable to local therapy.

*Conclusions*

A high proportion of patients with breast cancer BrM are ineligible for clinical trials that mandate “stability” of BrM.

Trials for the 40% of patients who have BrM in the setting of stable extracranial disease are required, particularly among those with HER2 + MBC given the intracranial efficacy of established and emerging systemic therapies.

## 23. Poster Presentation

**Utilization of Systemic Therapies for Metastatic Renal Cell Carcinoma (mRCC) in the Canadian Health Care System**
Luisa Cardenas ^1^, Naveen Basappa ^2^, Sunita Ghosh ^2^, Aaron Hansen ^3^, Christian Kollmannsberger ^4^, Lori Wood ^5^, Daniel Heng ^6^, Jeffrey Graham ^7^, Georg Bjarnason ^8^, Neil Reaume ^9^, Simon Tanguay ^10^, Denis Soulieres ^11^, Vincent Castonguay ^12^, Eric Winquist ^13^, Michel Pavic ^14^, Rodney H. Breau ^15^, Aly-Khan Lalani ^1^
^1^ McMaster University, Hamilton, ON, Canada
^2^ Cross Cancer Institute, Edmonton, AB, Canada
^3^ Princess Margaret Cancer Centre, Toronto, ON, Canada
^4^ BC Cancer Agency, Vancouver, BC, Canada
^5^ Dalhousie University, Halifax, NS, Canada
^6^ Tom Baker Cancer Centre, Calgary, AB, Canada
^7^ University of Manitoba, Winnipeg, MB, Canada
^8^ Sunnybrook Health Sciences Centre-Odette Cancer Centre, Toronto, ON, Canada
^9^ The Ottawa Hospital Regional Cancer Centre, Ottawa, ON, Canada
^10^ McGill University, Montreal, QC, Canada
^11^ CHUM-Centre Hospitalier de l’Université de Montréal, Montreal, QC, Canada
^12^ Centre Hospitalier Universitaire Pavillon l′Hôtel-Dieu de Quebec, Quebec City, QC, Canada
^13^ London Regional Cancer Program-London Health Science Center, London, ON, Canada
^14^ Centre Hospitalier Universitaire de Sherbrooke, Sherbrooke, QC, Canada
^15^ University of Ottawa, Ottawa, ON, Canada
* Correspondence: luisa.cardenas@medportal.ca

*Background*

Over the last decade, standard of care therapies for mRCC have greatly evolved. However, the availability of these emerging options in global health care systems can vary. We sought to describe the integration and usage of systemic therapies for mRCC in Canada since 2011.

*Methods*

We included mRCC patients enrolled in the Canadian Kidney Cancer Information System (CKCis), which includes 14 academic centers across Canada, from January 2011 to December 2020. Baseline demographic and tumor characteristics were identified, and patients were stratified by line of therapy (1L to 4L+), year of therapy initiation, and type of therapy (VEGF targeted therapy (VEGF-TT), mTOR inhibitor, anti-PD(L)-1-based monotherapy, combination, or other). Descriptive statistics were used: mean (SD) for continuous variables and frequency (%) for categorical values.

*Results*

We identified 3613 patients with mRCC, of whom 1097 (30%) did not receive systemic therapy (active surveillance, metastasectomy, poor performance status). Among patients who received 1L systemic therapy and had complete clinical data (*n* = 2502): median age was 64 years, 74% were male, 65% were Caucasian, 75% had clear cell histology. IMDC risk scores (good, intermediate, poor) were 17%, 56%, and 27% respectively. Among patients who discontinued therapy, 1269/2175 (58%) received 2L, and 590/1057 (56%) received 3L. Distribution of systemic therapies are shown in Table 1 by timepoints reflecting the general integration of anti-PD(L)-1-based therapies (pre, 2011–2015; post, 2016–2020).

*Conclusions*

Within the context of Canada’s publicly funded health care system, the availability of standard mRCC therapies broadly reflects access from government-funded, clinical trial, and access programs sources. In an evolving therapeutic landscape, ongoing advocacy is required to continue to facilitate patient access to efficacious therapies.

## 24. Poster Presentation

**Delivery of Cancer Care Via An Outpatient Telephone Support Line: Oncology Nursing Perspectives on Quality and Challenges**
Hely Shah ^1^, Lisa Vandermeer ^2^, Fiona MacDonald ^3^, Gail Larocque ^4^, Shannon Nelson ^4^, Mark Clemons ^1^, Sharon McGee ^1^
^1^ Division of Medical Oncology, Department of Medicine, The Ottawa Hospital Cancer Center, The Ottawa Hospital, Ottawa, ON, Canada
^2^ Ottawa Hospital Research Institute, Ottawa, ON, Canada
^3^ Queensway Carlton Hospital, Ottawa, ON, Canada
^4^ The Ottawa Hospital, Ottawa, ON, Canada
* Correspondence: heshah@toh.ca

*Rationale*

Patient support lines (PSLs) help in triaging clinical problems and providing psychosocial support to patients and care givers as they navigate a complex multi-disciplinary oncology team. The benefits of effective PSLs include reduced morbidity and depression and increased functional capacity. While providing support and training to nursing staff who operate these lines is key, there is limited data on their experience to guide this.

*Methods*

We invited oncology nurses with experience on The Ottawa Hospital Cancer Centre (TOHCC) PSLs to participate in an anonymous, online survey. Measures collected included nursing experience, characteristics of questions addressed by the PSL, patient and nursing satisfaction with the service, common challenges faced, and initiatives to improve patient and nursing experience.

*Results*

Seventy-one percent (30/42) of eligible nurses responded to the survey. The most common disease site, stage, issue, and symptom addressed were breast cancer, metastatic disease, treatment-related toxicity, and pain, respectively. Despite the majority of nurses reporting personal and patient satisfaction with the care provided by the PSL, there was variance in the perceived appropriate use of PSL by physicians and patients. As such, fifty-nine percent (17/29) of nurses recommended redefining the responsibilities of the PSL to achieve its maximal potential. Eighty percent (24/30) of nurses denied experiencing a reduction in PSL call volumes with increased patient access to their electronic medical record. Sixty percent (18/30) of nurses reported that having TOHCC-specific management plans for common issues would improve their experience and the quality of care provided by the PSL.

*Conclusions*

There is need for TOHCC-standardized management algorithms for common issues addressed on the PSL, as well as increased physician and patient education to redefine the goals of the PSL to help address problems with high volume and inappropriate calls. These data represent a first step in a planned quality improved study for the TOHCC PSL, with future initiatives including patient surveys and implementation of quality improvement initiatives with patient and staff feedback.

## 25. Poster Presentation

**An Assessment of Extended Pembrolizumab Dosing and Outcomes in Advanced Non-Small-Cell Lung Cancer Patients during the COVID-19 Pandemic**
Gordon Taylor Moffat ^1^, Lilian Hanna ^1^, Wilma Hopman ^2^, Andrea S. Fung ^1^, Pierre-Olivier Gaudreau ^1,3^
^1^ Department of Oncology, Queen′s University
^2^ Department of Public Health Sciences, Queen’s University
^3^ Canadian Cancer Trials Group, Cancer Research Institute, Queen’s University
* Correspondence: gtmoffat@gmail.com

*Background*

The COVID-19 pandemic led to Cancer Care Ontario’s approval of extended dosing (ED) of pembrolizumab in the management of metastatic non-small-cell lung cancer (mNSCLC) as a means of reducing the risk of exposure through fewer visits to the cancer center. Pembrolizumab ED has been evaluated through pharmacokinetic studies and simulated, model-based analyses; however, clinical data are lacking. This study aims to evaluate real-world outcomes and safety of ED vs. standard dosing (SD) of pembrolizumab.

*Methods*

This retrospective cohort study included patients with mNSCLC (all histologies) from the Cancer Centre of Southeastern Ontario (Kingston, ON, Canada) who received at least one cycle of single-agent pembrolizumab with SD (2 mg/kg IV up to a maximum of 200 mg q3 weeks) or ED (4 mg/kg IV up to a maximum of 400 mg q6 weeks) from January 2018 to December 2020. Data were collected from a review of clinical, pathological, and radiological reports. Study outcomes included the proportion of patients alive at data cut-off, the median number of treatment cycles, immune-related adverse events (irAEs), and time to toxicity (TTT). Statistical analyses included chi-square, Mann–Whitney, and *t*-tests.

*Results*

Of the 90 patients evaluated, 18 (20%) switched to ED. The proportion of those alive at the data cut-off was significantly higher in the ED vs. SD group (94% vs. 26%; *p* < 0.01). The ED group also received more cycles of therapy (median = 19, with 11.5 cycles received before the switch) compared to the SD group (median = 3.5). The median TTT was 130 (ED) vs. 105 (SD) days, and the rate (44% vs. 32%; *p* = 0.407) and severity of irAEs (≥grade 3 events: 50% vs. 52%) were similar. A higher proportion of ED patients discontinued treatment due to toxicity (45% vs. 15%; *p* < 0.01), and 5/8 (62.5%) of the irAEs from the ED group occurred after switching (majority of events resulted in treatment discontinuation).

*Conclusions*

A greater proportion of ED patients was alive at the time of data cut-off, but this may reflect a selection bias as they were more likely switched considering disease stability (i.e., more cycles were received prior to switching). The rate and severity of irAEs were similar between the two groups; however, a high proportion of irAEs in the ED group occurred following the switch, which justified treatment cessation. These results are limited by small patient numbers, and the possible impact of ED on toxicity should be further validated in prospective cohorts.

